# Comparison of Faba Bean Protein Ingredients Produced Using Dry Fractionation and Isoelectric Precipitation: Techno-Functional, Nutritional and Environmental Performance

**DOI:** 10.3390/foods9030322

**Published:** 2020-03-11

**Authors:** Martin Vogelsang-O’Dwyer, Iben Lykke Petersen, Marcel Skejovic Joehnke, Jens Christian Sørensen, Juergen Bez, Andreas Detzel, Mirjam Busch, Martina Krueger, James A. O’Mahony, Elke K. Arendt, Emanuele Zannini

**Affiliations:** 1 School of Food and Nutritional Sciences, University College Cork, T12 YN60 Cork, Ireland; m.vogelsangodwyer@umail.ucc.ie (M.V.-O.); sa.omahony@ucc.ie (J.A.O.); e.zannini@ucc.ie (E.Z.); 2 Department of Food Science, University of Copenhagen, 1958 Frederiksberg C., Denmark; ilp@food.ku.dk (I.L.P.); marcel@food.ku.dk (M.S.J.); jens.sorensen@siccadania.com (J.C.S.); 3 Fraunhofer Institute for Process Engineering and Packaging, Giggenhauser Str. 35, D-85354 Freising, Germany; juergen.bez@ivv.fraunhofer.de; 4 IFEU-Institut für Energie-und Umweltforschung Heidelberg GmbH, Im Weiher 10, 69121 Heidelberg, Germany; andreas.detzel@ifeu.de (A.D.); mirjam.busch@ifeu.de (M.B.); martina.krueger@ifeu.de (M.K.); 5 APC Microbiome Ireland, University College Cork, T12 YT20 Cork, Ireland

**Keywords:** faba bean, protein, dry fractionation, isoelectric precipitation, functional properties, FODMAPs, antinutrients, nutrition, carbon footprint, life cycle assessment

## Abstract

Dry fractionated faba bean protein-rich flour (FPR) produced by milling/air classification, and faba bean protein isolate (FPI) produced by acid extraction/isoelectric precipitation were compared in terms of composition, techno-functional properties, nutritional properties and environmental impacts. FPR had a lower protein content (64.1%, dry matter (DM)) compared to FPI (90.1%, DM), due to the inherent limitations of air classification. Of the two ingredients, FPR demonstrated superior functionality, including higher protein solubility (85%), compared to FPI (32%) at pH 7. Foaming capacity was higher for FPR, although foam stability was similar for both ingredients. FPR had greater gelling ability compared to FPI. The higher carbohydrate content of FPR may have contributed to this difference. An amino acid (AA) analysis revealed that both ingredients were low in sulfur-containing AAs, with FPR having a slightly higher level than FPI. The potential nutritional benefits of the aqueous process compared to the dry process used in this study were apparent in the higher in vitro protein digestibility (IVPD) and lower trypsin inhibitor activity (TIA) in FPI compared to FPR. Additionally, vicine/convicine were detected in FPR, but not in FPI. Furthermore, much lower levels of fermentable oligo-, di- and monosaccharides, and polyols (FODMAPs) were found in FPI compared to FPR. The life cycle assessment (LCA) revealed a lower environmental impact for FPR, partly due to the extra water and energy required for aqueous processing. However, in a comparison with cow’s milk protein, both FPR and FPI were shown to have considerably lower environmental impacts.

## 1. Introduction

The important role of plant proteins in human nutrition is becoming increasingly recognized. More sustainable options than animal protein are needed in order to guarantee food security, as the global population is projected to increase by two billion over the next 30 years [1,2]. The conversion of feed protein to animal proteins is inherently inefficient, and plant protein crops such as legumes, which are mainly used as feed, may be better utilized for direct human consumption [3]. Additionally, animal proteins, including dairy, meat, and eggs, are generally associated with greater environmental impacts compared to plant proteins [4], therefore, further research in the area of plant-based substitutes for these should be prioritized. Faba beans (*Vicia faba*) provide a good source of sustainable plant protein and are currently underutilized [5]. However, pulses are known to contain antinutrients such as trypsin inhibitors, which decrease their nutritional value. Faba beans in particular also contain vicine and convicine; these natural pyrimidine glycosides are known to induce the disease favism in certain individuals who have an inherent deficiency in glucose-6-phosphate dehydrogenase, thereby restricting the usage of faba bean ingredients in foods [6,7]. Additionally, galacto-oligosaccharides (GOS) are found in high amounts in faba beans [8]. GOS are included in the Fermentable Oligo-, Di- and Monosaccharides And Polyols (FODMAP) family. FODMAPs are poorly digestible, and are fermented and/or osmotically active in the distal small intestine and proximal colon, thus can lead to increased fluid as well as gas production [9]. Adhering to a low FODMAP diet has been shown to provide symptomatic relief to irritable bowel syndrome (IBS) sufferers [10], and such diets can be personalized depending on the individual [11]. A number of methods have been used to produce high-protein ingredients from pulses. Protein isolates may be produced using aqueous extraction methods; these include alkaline, neutral, or acid extraction, followed by isoelectric precipitation (IEP) or ultrafiltration (UF), as well as salt extraction followed by micellization [12,13]. Another option to produce protein ingredients is dry fractionation, where dried legumes are milled, and the particles are then air classified based on size and density into protein-rich and starch-rich fractions [14]. A number of advantages of dry fractionation compared to aqueous processing have been observed, including a significantly lower use of energy and water, as well as the preservation of the native protein functionality [14,15,16]. On the other hand, there are also disadvantages to this approach, which include lower protein purity, as well as undesirable components such as antinutrients and oligosaccharides not being effectively removed [6,8,14]. If plant proteins are used to provide alternatives to animal-based products, they should provide certain functional properties. The objective of this study was to compare two novel protein ingredients derived from faba bean, namely faba bean protein-rich flour (FPR) and faba bean protein isolate (FPI), which were produced using two different processing regimes, by analyzing various physicochemical, techno-functional and nutritional properties of relevance for food applications. Additionally, life cycle assessment (LCA) was employed to assess the environmental impacts associated with their production. 

## 2. Materials and Methods 

### 2.1. Chemicals and Raw Materials

Chemicals were purchased from Sigma-Aldrich (St Louis, MI, USA), unless otherwise stated. Faba beans (*Vicia faba* L. cv. Imposa) were delivered by Louis Bolk Institute (The Netherlands). This no-tannin variety was chosen as it was expected to be low in vicine/convicine. 

### 2.2. Preparation of Faba Bean Protein Ingredients

#### 2.2.1. Faba Bean Protein-Rich Flour 

Faba beans were dehulled in an underrunner disc sheller, with subsequent separation of the hulls from the kernels using a zigzag classifier. The resulting kernels were fine milled (d90 = 30 µm) using a 200 ZPS classifier mill (Hosokawa-Alpine, Augsburg, Germany), adjusted to a mill speed of 5300 rpm. During milling, an internal classifier wheel allowed fine particles to leave the grinding chamber, while coarse particles were recirculated. In the next step, the resulting fine particles were passed into a Turboplex 200 ATP air classifier (wheel speed 5800 rpm) to separate the smaller protein-rich fragments from larger starch granules or fiber-rich particles. 

#### 2.2.2. Faba Bean Protein Isolate 

FPI was the outcome from a single-batch pilot scale processing starting with a dehulled faba bean fraction, utilizing a patented aqueous extraction method [17]. In brief, a dehulled faba bean fraction was wet milled under heated acidic conditions and then fibers and insoluble proteins were removed using centrisieve technology. Starch was separated from the protein slurry by means of decantation (Foodec 200; Alfa Laval, Nakskov, Denmark), followed by IEP at pH 4.8 to produce the protein isolate separated by decantation. The precipitated proteins were subsequently adjusted to pH 6.8, and a slurry of the protein isolate fraction was dried using a Mobile Minor pilot scale spray dryer (GEA Niro, Søborg, Denmark).

### 2.3. Compositional Analysis

Compositional analysis was carried out by Concept Life Sciences Ltd. (Manchester, UK) using the following methods: protein content was analyzed using the Dumas method using a nitrogen-to-protein conversion factor of 6.25; fat content was measured using low resolution proton nuclear magnetic resonance; saturated, mono-unsaturated, poly-unsaturated and trans fatty acids were quantified using gas chromatography–flame ionization detection (GC-FID) analysis; ash content was determined by oxidation at 550 °C to remove organic matter; moisture was determined by oven drying (105 °C) for a minimum of 16 h; sodium was determined using flame photometry after ashing at 550 °C; other minerals were analyzed using inductively coupled plasma atomic emission spectroscopy or ion chromatography. Total carbohydrate content was calculated by difference. Amino acid composition was determined by Chelab S.r.l. using ion chromatography with post-column derivatization with ninhydrin, or HPLC-UV analysis in the case of tryptophan.

### 2.4. Protein Profile Analysis

An Agilent Bioanalyzer 2100 Lab-on-a-Chip capillary electrophoresis system was used to analyze the protein profile and estimate the molecular weights of the respective protein bands. Samples were prepared according to Amagliani et al. [18] with slight modifications: protein ingredients were dispersed in 2% SDS, 2 M thiourea and 6 M urea, to give a protein concentration of 2.5 mg/mL. Dispersions were shaken for 2 h at 22 °C, and centrifuged to remove insoluble material. Samples were analyzed using an Agilent Protein 80 kit and Protein 230 kit according to the instructions within the ranges of 5–80 and 14–230 kDa, respectively. For reducing conditions, dithiothreitol (DTT) was included in the sample buffer according to kit instructions.

### 2.5. Scanning Electron Microscopy 

Scanning electron microscopy (SEM) was carried out according to the method of Alonso-Miravalles et al. [19] using a JSM-5510 scanning electron microscope (JEOL Ltd, Tokyo, Japan).

### 2.6. Particle Size Distribution

The particle size distribution (PSD) of protein dispersions was measured using a static laser light diffraction unit (Mastersizer 3000, Malvern Instruments Ltd, Worcestershire, UK), covering a size range of 0.01–3000 μm. For the preparation of samples, protein ingredients were dispersed in ultrapure water in 50 mL centrifuge tubes at a concentration of 1% protein (*w/v*), pH adjusted to 7, and samples were shaken overnight at 4 °C. The particle refractive index was set at 1.45, the absorption used was 0.1 and the dispersant refractive index was 1.33. Protein dispersions, equilibrated to 22 °C, were introduced into the dispersing unit using ultrapure water as dispersant, until a laser obscuration of 12% was achieved. Results were presented as volume-weighted mean particle diameter (D[4,3]), surface-area weighted mean particle diameter (D[3,2]) and volume percentiles (Dv(10), Dv(50), and Dv(90)). 

### 2.7. Surface Hydrophobicity 

Surface hydrophobicity (S_0_) was measured based on the method of Hayakawa and Nakai [20], using 1-anilino-8-naphthalenesulfonate (ANS) with slight modifications as described by Karaca et al. [21] Protein dispersions were serially diluted with 10 mM phosphate buffer (pH 7) in the range of 0.0006–0.015% (*w/v*). ANS (10 µL; 8.0 mM in 0.1 M phosphate buffer, pH 7) was mixed with a 2 mL diluted sample and left in darkness for 15 min. Fluorescence was measured (λ_excitation_ 390 nm, λ_emission_ 470 nm) and corrected by a blank measured without ANS. The results are presented as the slopes (*R*^2^ ≥ 0.98) of the absorbance versus protein concentration.

### 2.8. Protein Solubility

Protein solubility, as influenced by pH, was evaluated. First, the protein content (N×6.25) of protein ingredients was measured using the Kjeldahl method, dispersions of 1% (*w/v*) protein were prepared, and the pH was adjusted from 3.0 to 8.0 in 0.5 pH unit intervals using HCl or NaOH. Dispersions were hydrated at 4 °C overnight. Samples were then adjusted to 22 °C while shaking and pH was re-adjusted if necessary. Samples were centrifuged at max. speed (4893× *g*) for 30 min and the protein contents of the resultant supernatants were also measured using the Kjeldahl method. Protein solubility was expressed as a % of original protein content in the dispersion remaining in the supernatant.

### 2.9. Zeta Potential 

The zeta potential of protein dispersions over the same pH range as for protein solubility analysis were determined using a Zetasizer nano-Z (Malvern Instruments Ltd; UK). Samples (0.1% protein *w/v*) were prepared in ultrapure water and pH was adjusted using HCl or NaOH. Samples were shaken overnight at 4 °C, adjusted to 22 °C and pH was readjusted if necessary. Samples were then centrifuged at 2000× *g* for 10 min to remove any insoluble material. The measurement was performed using an automatic voltage selection and zeta potential was calculated using the Smoluchowski model. A refractive index and absorption of 1.45 and 0.001 were used, respectively.

### 2.10. Fat Absorption Capacity

Fat absorption capacity (FAC) was determined following the method described by Boye et al. [22] with slight modifications. Powder (1 g) and sunflower oil (6 g) were weighed into a 15 mL centrifuge tube, mixed with a vortex for 3 min, and centrifuged at 4000× *g* for 30 min. The oil was removed from the tube carefully and weighed again. FAC was expressed as grams of fat retained per 100 g protein ingredient.

### 2.11. Foaming Properties

Foaming properties were assessed according to the method of Alonso-Miravalles et al. [19] Dispersions (20 mL in 50 mL centrifuge tubes) with a protein concentration ranging from 0.1 to 3.3% (*w/v*) in 0.1 M phosphate buffer pH 7 were frothed using an Ultra-Turrax equipped with a S10N-10G dispersing element (Ika-Labortechnik, Janke and Kunkel GmbH, Staufen) at maximum speed for 30 s. The height of the sample (liquid and foam phase) was measured immediately, and after 60 min. Foaming capacity was taken as % sample expansion at 0 min, while foam stability was taken as sample expansion at 60 min as a percentage of sample expansion at 0 min. Sample expansion was calculated using the following equation:

Sample expansion (%) = ((Sample height after foaming − initial sample height)/Initial sample height)·100

### 2.12. Minimum Gelling Concentration

Minimum gelling concentration of each protein was determined using protein dispersions in 10 mM phosphate buffer (pH 7) in the range of 5–15% protein. Dispersions (5 mL) were prepared in 15 mL centrifuge tubes and hydrated overnight at 4 °C. Tubes were heated at 90 °C in a water bath for 30 min, cooled rapidly under running water, and maintained overnight at 4 °C. Tubes were then inverted and the minimum protein concentration at which the dispersion did not flow was taken as minimum gelling concentration.

### 2.13. Rheological Analysis of Heat Gelation Properties

Rheological tests were carried out using a controlled stress rheometer (MCR301, Anton Paar GmbH, Austria) equipped with a concentric cylinder measuring system (C-CC27-T200/SS, Anton Paar GmbH, Austria). Protein dispersions (10 or 15% *w/v*) were hydrated overnight at 4 °C, adjusted to 22 °C, sheared for 10 s at speed 3 with an Ultra-Turrax T10 equipped with a S10N-10G dispersing element (Ika-Labortechnik, Janke and Kunkel GmbH, Staufen) to ensure there were no lumps, and pH was then adjusted to 7.0. Small deformation oscillatory rheology was used to monitor heat gelation with strain and frequency of 0.1% and 1 Hz, respectively. The temperature profile used was as follows: temperature was increased from 20 to 90 °C at 2 °C/min, held at 90 °C for 30 min, cooled to 20 °C at 2 °C/min, and held at 20 °C for 30 min. This was followed by a logarithmic frequency sweep from 0.01 to 10 Hz, maintaining strain at 1%. Following this, the large deformation properties of the gels were examined by applying rotational shear at a shear rate of 0.005 s^−1^, and stress-strain curves were generated [23], from which the gel fracture properties could be assessed.

### 2.14. In Vitro Protein Digestibility

Gastro-pancreatic protein digestion was simulated using a multistage static in vitro protein digestibility (IVPD) method, as described previously [24,25]. In addition to FPR and FPI, a faba bean dehulled flour (FDH) from the same faba bean source was also analyzed for comparison. Briefly, faba bean ingredients were initially standardized to contain 50 ± 0.1 mg protein on a DM (dry matter) basis. Enzymatic hydrolysis was performed by pepsin digestion (1 h, 37 °C), followed by sequential pancreatin digestion in the short- (1 + 1 h, 37 °C), medium- (1 + 3 h, 37 °C), and long-term (1 + 24 h, 37 °C) stages. Enzyme to substrate (E:S) ratios during pepsin and pancreatin digestion stages were maintained constant, at about 1:50 and 1:10 *w/w*, respectively. IVPD was calculated as the ratio between the concentration of free α-amino groups in the samples and the alanine standard solution using a trinitrobenzenesulfonic acid (TNBS) based method, with the alanine standard representing 100% protein digestibility.

### 2.15. Trypsin Inhibitor Activity (TIA) Assay

Trypsin inhibitors were initially extracted from the faba bean ingredients (including FDH) by diluting the samples (350 mg) in sodium acetate buffer (2.5 mL, 0.1 M, pH 4.9), followed by homogenization by Ultra-Turrax for 2 min. The samples were centrifuged for 5 min at 3000× *g* (EBA 12 Centrifuge; Hettich Zentrifugen, Tuttlingen, Germany) and the supernatant was transferred into a new test tube. The procedure was repeated under the same conditions, where the residual pellet was resolubilized, homogenized, and centrifuged. The two supernatants were pooled, kept in the refrigerator overnight, and centrifuged again for 5 min at 3000× *g* prior to TIA analysis. TIA levels of the faba bean ingredients were determined using a previously described method, with a few modifications [25]. In short, the TIA levels were determined against a trypsin enzyme solution with a stock concentration of 0.1 mg/mL. The substrate solution used was 0.22 mg/mL N-α-benzoyl-l-arginine-4-nitroanilide (l-BAPA). A molar extinction coefficient for the product (4-nitroaniline) of 8800 M^−1^ × cm^−1^ was used for spectrophotometric quantification at 410 nm. In this assay, 1 trypsin inhibitor unit (1 TIU) is defined as the amount of inhibitor required to reduce the enzyme activity by 1 trypsin activity unit (TU). TU is defined as the amount of enzyme that catalyzes hydrolysis of 1 μmol of l-BAPA into 4-nitroaniline in 1 min at pH 8.2 at 37 °C. TIA levels of the samples were calculated based on dry sample mass or dry protein mass, and expressed as TIU/mg sample DM or TIU/mg protein DM.

### 2.16. Vicine and Convicine Analysis

The content of vicine and convicine in the faba bean ingredients (including FDH) was analyzed in a crude extract prepared according to Petersen et al. [26], and modified by adding 50 µL internal standard (189.9 mM trigollinamide) to 500 mg faba bean sample, prior to extraction with boiling methanol as described. The resulting crude extract was analyzed for the contents of vicine and convicine using micellar electrokinetic capillary chromatography (MECC), as described by Bjergegaard et al. [27] The concentrations of vicine and convicine were calculated based on dry sample mass or dry protein mass (by correcting for the dry matter and protein contents), using an internal TNA standard and an external vicine standard, and expressed as mg/g sample DM or mg/g protein DM.

### 2.17. Quantification of Fermentable Oligo-, Di- and Monosaccharides, and Polyols (FODMAPs)

The quantification of mono-, di-, galactooligosaccharides, fructans, and polyols was conducted using high performance anion-exchange chromatography coupled with pulsed amperometric detection (HPAEC-PAD), performed on a DionexTM ICS-5000+ system (Sunnyvale, CA, USA), as described by Ispiryan et al. [28] All carbohydrates, except for the fructans, were quantified using authentic reference standards, as specified in the previous study [28]. The total fructan content was determined after enzymatic hydrolysis with two enzyme mixtures A and B, where only B contained fructan degrading inulinases. The calculation was based on the quantification of the monomers glucose and fructose released from the fructan molecules [28]. The significance of the fructose released from sucrose and the fructose released from the hydrolysis with the enzyme mixture B was determined; if no significant difference was determined and all levels below 0.1 g/ 100 g are referred to as not detected (n.d.) in further discussions [29]. All extractions were carried out in duplicate, according to the method described by Ispiryan et al. [28] The results of the ingredients (including FDH) are presented as g analyte per 100 g sample on a dry weight basis (g/100 g DM). 

### 2.18. Life Cycle Assessment 

Environmental performance of faba bean protein ingredients was examined by means of LCA using Umberto 5.5 software (ifu Institut für Umweltinformatik GmbH, Hamburg, Germany). The assessment was carried out as an attributional cradle-to-gate LCA and includes the individual processes associated with faba bean protein ingredients. Impact assessment methods were based on Detzel et al. [30].

### 2.19. Statistical Data Analysis

Unless otherwise stated, all analyses were carried out in triplicate, with the exception of compositional analyses, which were performed following validated methods and therefore analyzed only once and reported without standard deviation. In the case of the amino acid (AA) analysis, validated uncertainty values are included. Results were subjected to two-tailed, unpaired Student’s t-test to determine statistically significant differences (*p* < 0.05) between mean values for the different samples, or one-way ANOVA followed by Tukey’s post hoc test for IVPD and TIA analysis. The statistical programs used were IBM SPSS version 26 (Armonk, USA) or GraphPad Prism version 8.3.0. (San Diego, CA, USA) The results are presented as mean ± standard deviation where applicable. 

## 3. Results and Discussion

### 3.1. Compositional Analysis

Nutritional composition of FPR and FPI is shown in Table 1. A higher protein content was measured for FPI (90.1%, DM) compared to FPR (64.1%, DM). Typically, air classification of plant proteins results in lower protein purity than is obtained by aqueous extractions. This is due to the physical limitations of the process in separating protein bodies from starch granules and other seed material, and the protein content of the protein bodies is a limiting factor [12,31]. Protein contents reported in the literature for air classified faba bean protein-rich fractions have been in the range of ~50–70% (DM) [16,32,33,34]. Protein contents of similarly produced protein-rich fractions from peas are typically in the range of ~50–60% (DM) [35,36]. On the other hand, aqueous processing allows for higher protein purity; faba bean and other legume protein isolates are often in the range of 80–90% or higher [12,37,38]. Fat content was higher in FPI than FPR, indicating that fat was concentrated to some extent as part of the isolation process. However, the ratio of saturated, mono- and polyunsaturated fatty acids was similar for both ingredients. The ash content was slightly higher for FPI, and sodium content was also higher. This may be due to the addition of alkaline solution to raise the pH of the protein isolate prior to drying [19]. Starch content and total carbohydrate by difference were higher in FPR, indicating that the aqueous isolation process was more effective than the dry fractionation for starch removal. In the case of FPR, Coda et al. [32] reported higher starch content (23.38%) for an air classified faba bean protein-rich flour, albeit with lower protein content. Differences in composition may be due to different starting materials, processing, and equipment. 

### 3.2. Structural and Surface Properties

#### 3.2.1. Protein Profile

**Figure 1:**
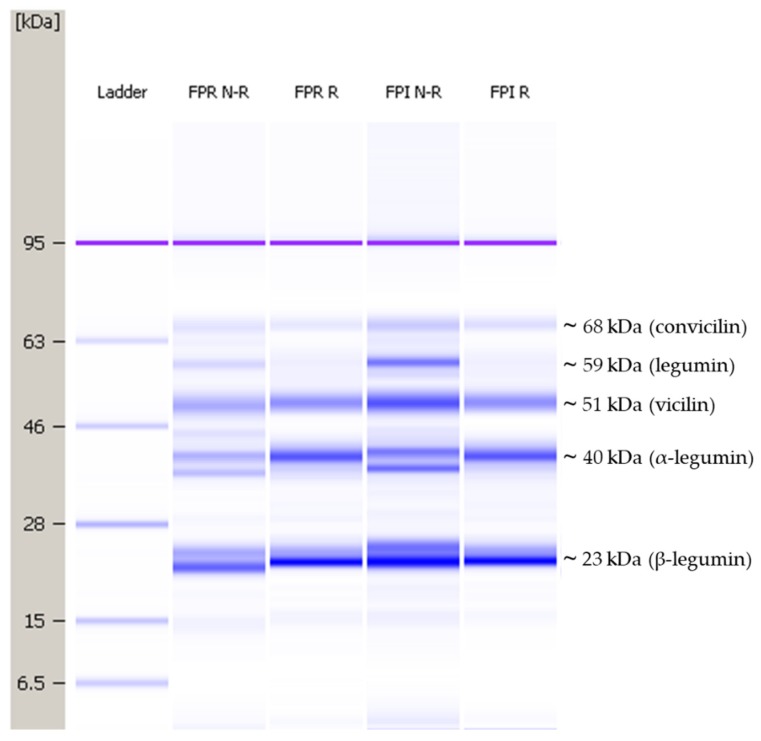
Representative protein profiles for faba bean protein-rich flour (FPR) and faba bean protein isolate (FPI) under non-reducing and reducing conditions, in the range of 5–80 kDa. N-R: non-reducing conditions, R: reducing conditions.

Protein profiles under reducing and non-reducing conditions are shown in Figure 1. The profile was quite similar to previously published SDS-PAGE results for faba bean protein [34,37,39]. The majority of proteins found in pulses are globulins (~70–78%), followed by albumins (10–20%) [1,40]. The bands visible at ~68, ~59, and ~51 kDa likely correspond to subunits of the globulins convicilin, legumin, and vicilin, respectively. Legumin subunits are composed of an acidic (α-legumin) and basic (β-legumin) chain linked by a single disulfide bond; the bands around 40 kDa and 23 kDa are expected to be α- and β-legumin, respectively. The band at ~59 kDa was present for both FPR and FPI under non-reducing conditions but was absent under reducing conditions. This indicates the further dissociation of legumin into its acidic and basic subunits under reducing conditions. Pulse protein isolates produced by IEP are typically more enriched in globulins, as the albumin fraction retains solubility around the pI of globulins [21]; however, no major differences in protein composition were apparent between FPR and FPI from the protein profile. 

#### 3.2.2. Scanning Electron Microscopy

**Figure 2:**
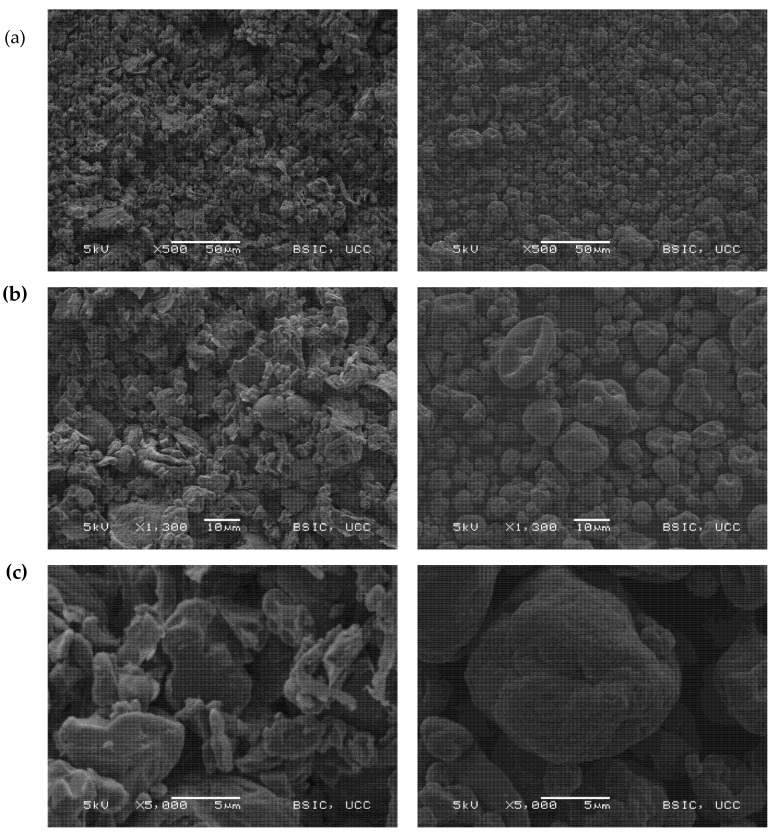
Representative scanning electron micrographs of FPR (left column) and FPI (right column). Magnifications shown are (**a**): 500×, (**b**): 1300× and (**c**): 5000×.

SEM micrographs of the dry ingredients (Figure 2) depict the differences in morphology between FPR and FPI. In FPR, a small number of smooth round starch granules (~10–20 µm) can be seen, surrounded by irregularly-shaped particles of seed material which are assumed to consist mainly of protein bodies, along with cell wall fragments [35,41]. FPI, by contrast, has the typical appearance of spray dried protein powders—smooth, rounded, shrunken particles resulting from evaporation of water from the liquid feed droplets during drying [19,42]. In the case of FPR, due to the uneven and clustered nature of the particles, it is more difficult to identify discrete particles compared to FPI. 

#### 3.2.3. Particle Size Distribution of Dispersions

**Figure 3:**
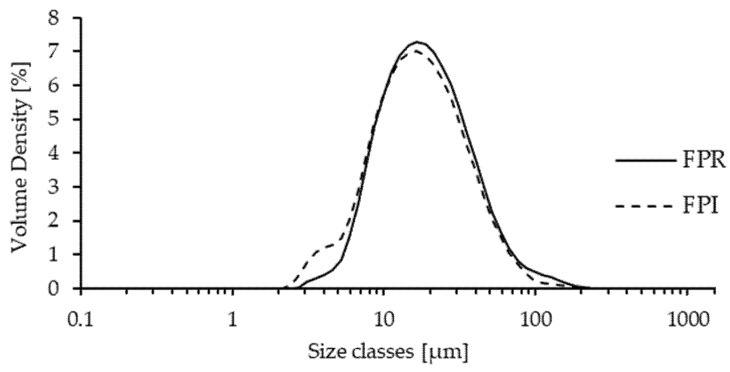
Volume weighted particle size distribution for FPR and FPI after overnight hydration at 4 °C, showing volume density (%) as a function of size class (μm).

The volume-weighted particle size distributions for FPR and FPI dispersions are shown in Figure 3. Both distributions are monomodal and occupy a very similar range, ~2–200 µm. Volume weighted mean particle diameter (D[4,3]) was 25.4 µm for FPR and 22.9 µm for FPI (Table 2). No significant differences were found between FPR and FPI for D[4,3], Dv(50) or Dv(90). Significantly lower D[3,2] and Dv(10) values were found for FPI compared to FPR, suggesting a slightly greater prevalence of smaller particles, although the differences were not major. The size range observed for FPR and FPI corresponds approximately with those found for spray dried lentil protein isolate and milk protein concentrate powders in aqueous dispersions [19,43], as well as the dry particle size of air classified pea protein-rich flour [41].

#### 3.2.4. Surface Hydrophobicity 

Surface hydrophobicity (S_0_) of proteins is an important property which influences their functionality including solubility, foaming, and emulsifying properties [21,44]. S_0_ was found to be significantly higher for FPI compared to FPR. In the native structure of globular proteins in water, hydrophobic regions tend to be buried in the internal folded regions to minimize free energy [45]. However, the changes in pH and high temperatures utilized during the isolation and spray drying of FPI may have led to denaturation, exposing previously buried hydrophobic regions, resulting in the higher measured S_0_. FPR, on the other hand, may have retained a more native protein structure due to the milder processing conditions of dry fractionation [35].

### 3.3. Techno-Functional Properties 

#### 3.3.1. Protein Solubility and Zeta Potential

**Figure 4:**
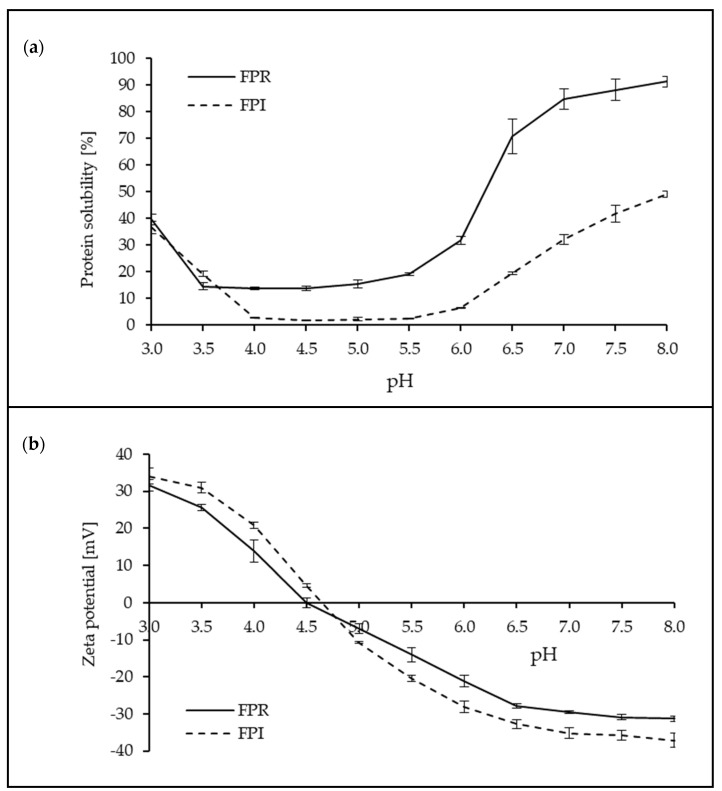
Protein solubility (%) (**a**) and zeta potential (mV) (**b**) as a function of pH for FPR and FPI (error bars show + one standard deviation).

Protein solubility is an important property for many food systems, and good solubility is often required for other functional properties, such as foaming and gelation [1,46]. The ability of proteins to remain solubilized depends on the balance between protein–protein and protein–water interactions and surface charge is an important factor influencing protein solubility. Similarly charged particles repel each other, limiting protein–protein interactions, and promoting protein–water interactions, thus allowing them to remain solubilized [21,40,44]. The inter-relationships between pH, surface charge, and protein solubility can be clearly seen in Figure 4. The isoelectric point (where net charge is zero) was observed at pH ~4.5 for both FPR and FPI, corresponding to minimum solubility. For FPR the lowest solubility was observed around pH 3.5–5.5, and for FPI around pH 4–6. Although the overall pattern was similar for both ingredients, protein solubility was generally lower for FPI over the pH range studied. Solubility of FPR was more than double that of FPI at neutral pH. This difference was apparently not related to surface charge, as zeta-potential values were similar. However, there are several potential reasons for this difference. For example, denaturation during the isolation and drying process may be responsible for increased hydrophobicity in FPI, which could contribute to lower solubility. Heating has been shown to have a negative effect on protein solubility in faba bean flour [46]. Additionally, the spray dried protein particles of FPI may present difficulties in terms of rehydration, something which has been observed in milk protein concentrate powders [43], where a ‘skin’ at the particle surface can retard solubilization. In literature, protein solubility values for IEP faba bean protein at neutral pH vary from ~24% to 85% [13,38,47], generally higher than the FPI examined in this study. However, differences in extraction pH, drying methods, and indeed the methods used to determine solubility, may have contributed to the observed differences in solubility. The higher protein solubility of FPR compared to FPI may provide an advantage in high-protein beverage type applications such as milk substitutes, where good solubility is required. 

#### 3.3.2. Foaming Properties

**Figure 5:**
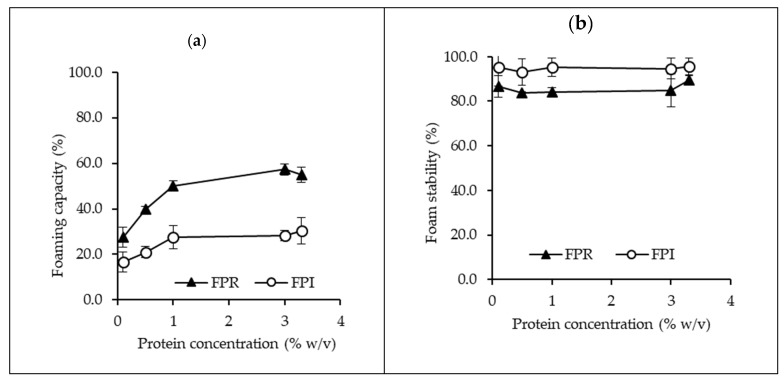
Foaming capacity (**a**) and foam stability (**b**) for FPR and FPI in the range of 0.1–3.3% protein, at pH 7 and 22 °C (error bars show standard deviation).

The foaming capacity and foam stability for FPR and FPI in the range of 0.1–3.3% protein are shown in Figure 5. For both FPR and FPI the foaming capacity increased with protein concentration from 0.1–1%, with further increase in concentration having a minimal effect. At all concentrations measured, foaming capacity was significantly greater for FPR compared to FPI. Foam stability was similar across the concentration range. The lower solubility of FPI may have contributed to its lower foaming capacity [48,49]. Additionally, a higher proportion of albumins in FPR compared to FPI may have contributed to the higher foaming capacity [50], although this was not apparent in the protein profile (Figure 1).

#### 3.3.3. Fat Absorption Capacity

FAC is often related to emulsifying capacity and is an important property for certain food applications, such as mayonnaise, meat and dairy-type products [51,52]. FAC was 124 ± 5.8 and 87.2 ± 2.5 g/100 g for FPR and FPI, respectively. Vioque et al. [53] reported higher FAC for faba bean protein isolate compared to faba bean flour, possibly due to increased exposure of hydrophobic groups due to denaturation in the isolate, however, FAC was lower for FPI in this study. FPR had comparable FAC (>100 g/100 g) to lentil, pea and chickpea protein concentrates produced using IEP [22].

#### 3.3.4. Gelation Properties

Protein gelation is an important functional property for various food applications; cheese, yoghurt and tofu are examples of such foods where protein gelation is critical. For globular proteins, heat-induced gelation requires denaturation, aggregation, and an above critical concentration, formation of a continuous protein network [54]. Non-covalent (electrostatic, hydrophobic interactions, and hydrogen bonds) and covalent interactions (disulfide bonds) may be involved in gelation [55,56]. The minimum gelling concentrations determined for FPR and FPI were 7% and 12% protein, respectively. 

**Figure 6:**
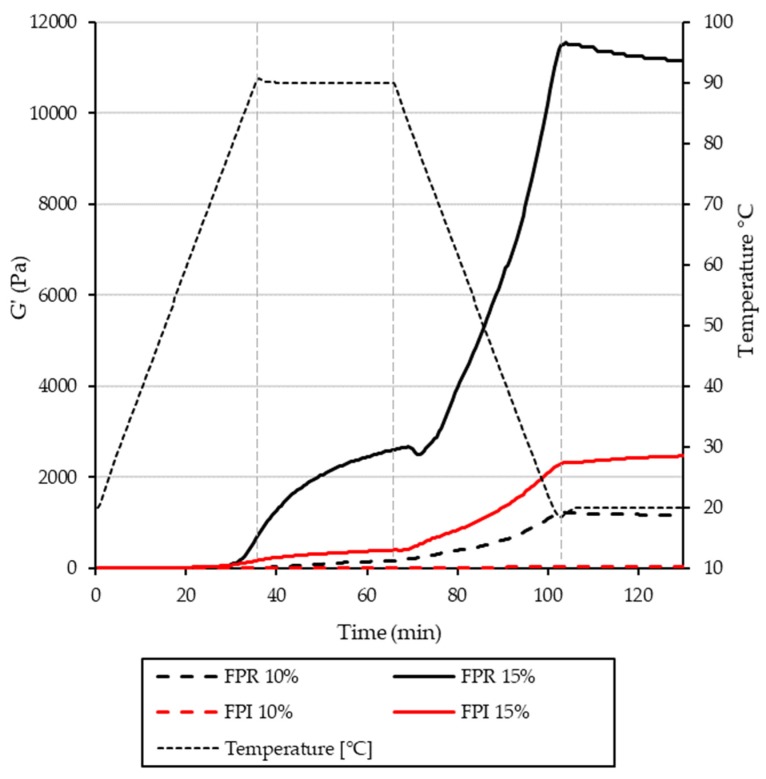
Rheological characterization of gel formation during heating and cooling. Storage modulus (Pa) and temperature (°C) are shown as a function of time for FPR and FPI at 10% and 15% protein.

**Figure 7:**
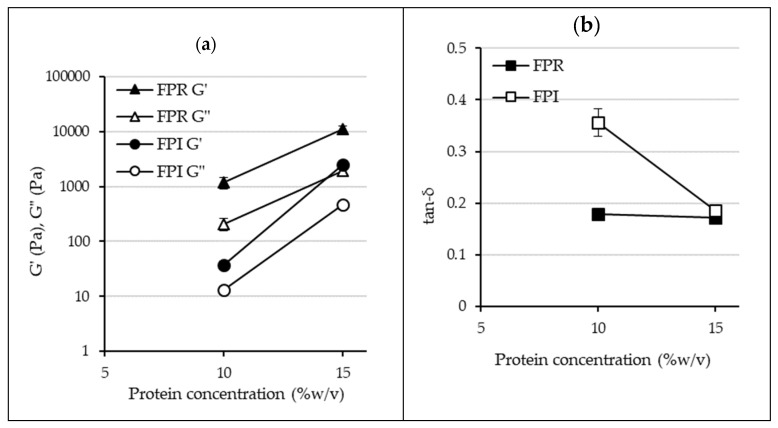
Final values for G’ and G” (**a**), and tan-δ (**b**) for FPR and FPI at 10% and 15% protein (error bars show standard deviation).

This difference in gelling behavior was also reflected in the rheological heating/cooling cycle (Figure 6 and Figure 7). Final G’ values were higher for FPR at both 10% and 15% protein. In the case of both FPR and FPI, gel strength increases with protein concentration. The overall pattern observed during heating and cooling was similar to that observed for other plant proteins including pea, soy cowpea, and quinoa [57,58,59,60]. This involved an initial increase in G’ during heating, followed by a greater increase during cooling as the gel network strengthened. Final tan-δ values were <0.2 for FPR at 10% and 15%, and for FPI at 15% protein, revealing the elastic nature of the structures formed after heating and cooling (Figure 7). However, tan-δ for FPI at 10% protein was 0.36, indicating less elastic behavior, which was also reflected in its inability to form a self-supporting gel at this concentration. Two different measurements were used to describe the onset temperature of gelation: G’>G” crossover point (where the elastic response becomes greater than the viscous response), and the temperature at which the rate of increase in G’ reached 0.5 Pa/°C [61] (Table 3). It can be seen that the onset of gelation occurred at lower temperatures for FPI than FPR, even though the final gels were weaker. At least for FPR, it was apparent that this onset temperature became lower as concentration increased; this trend has also been observed with soy proteins [61]. The initial gel formation of legume proteins is most likely due to denaturation of 7S globulin (vicilin), and G’>G” crossover temperatures averaging 64.1 °C have been observed for Kabuli chickpea protein, as well as 67.5 °C for a soy protein isolate [62] at 12% protein.

**Figure 8:**
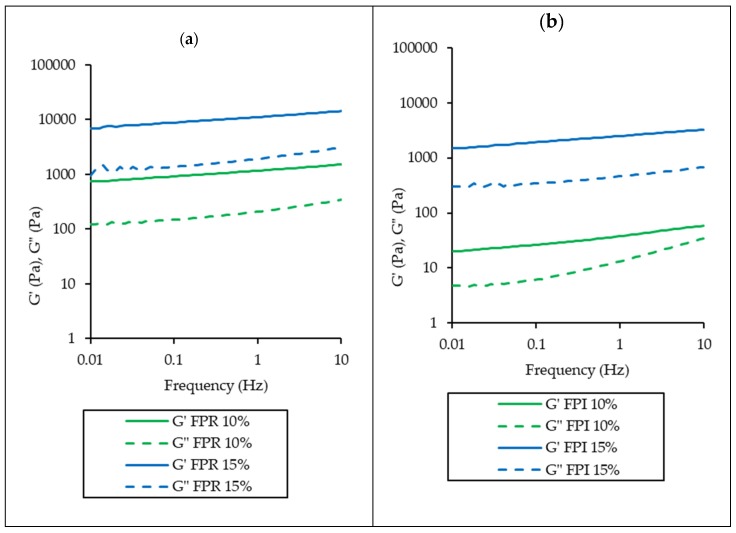
Rheological frequency sweeps for FPR (**a**) and FPI (**b**) at 10% and 15% protein. Storage modulus (Pa) and loss modulus (Pa) are shown as a function of frequency (Hz).

The mechanical spectra for FPR and FPI are shown in Figure 8, which also shows the greater gelling ability of FPR compared to FPI. For all samples, G’ was greater than G” over the frequency range, indicating gel formation [63]. In the case of FPR 10% and 15%, as well as FPI 15%, G’ and G” values show relatively little dependence on frequency, suggesting a stronger gel, while FPI 10% displays more frequency dependence and more viscous behavior at higher frequency, indicating a weaker gel structure [64].

**Figure 9:**
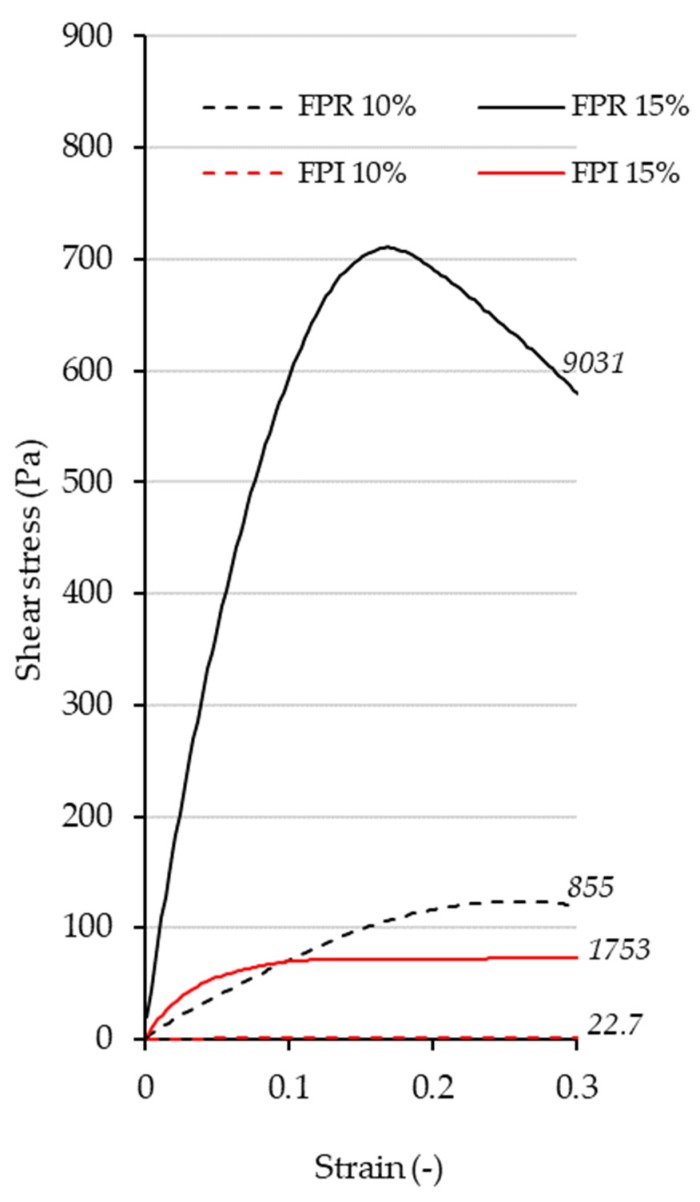
Rotational stress-strain curves for FPR and FPI and 10% and 15% protein with shear stress (Pa) shown as a function of strain (-). Young’s modulus (Pa) is shown for each curve in italics.

The large deformation properties for FPR and FPI gels are shown in Figure 9. Large deformation properties of foods are important in relation to production, handling, and the consumer’s experience during the preparation and eating of foods [50,61]. It can be seen that FPR produced stronger gels at both protein concentrations measured. A clear fracture point was only apparent for FPI at 15% protein, whereas for the other curves, stress appears to level off as strain increases, indicating softer/viscous structures. Young’s modulus (initial slope) was also higher for FPR than FPI at both concentrations, which also corresponds to the small deformation results. The higher solids content at a given protein concentration of FPR compared to FPI may have contributed to its greater gelling ability. Additionally, in the case of pea protein gels, the level of starch has been shown to have a major effect on gel firmness [15]. The higher protein solubility of FPR compared to FPI may also have been a contributing factor [14]. FPR may be more suitable for applications where greater gel strength is desired, whereas FPI may be more suitable if a softer gel is necessary, or if higher protein purity is required.

### 3.4. Nutritional Properties

#### 3.4.1. Amino Acid Profile

The AA profiles of FPR and FPI are shown in Table 4. There were slight differences between FPR and FPI, indicating that the protein composition was not altered considerably during the IEP procedure. The AA profiles compare well to those found in the literature for faba bean protein ingredients [8,47].

**Figure 10:**
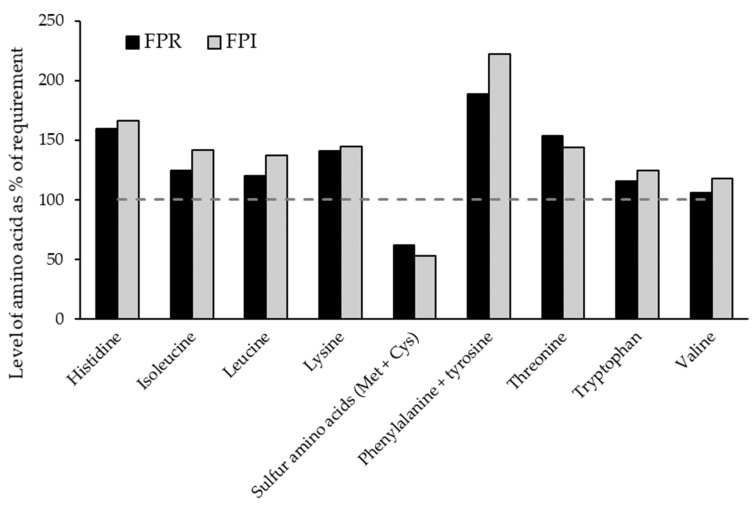
Levels of indispensable/conditionally indispensable amino acids in FPR and FPI, as a percentage of the World Health Organization (2007) adult requirement for each amino acid [65].

The indispensable amino acid (IAA) content as a percentage of the WHO (2007) adult requirements [65] are shown in Figure 10. The levels were similar, although the level of most IAAs was slightly higher for FPI, with the exceptions of sulfur-containing AAs (SAAs) and threonine. All AA were above the requirement threshold except SAAs, which is unsurprising, as faba beans as well as other legumes typically tend to be low in SAAs. Expressing the limiting AA content (in this case SAA) as a fraction of the WHO adult requirement results in AA scores of 0.62 for FPR and 0.53 for FPI. The reason for this slight difference may be a loss of sulfur-rich albumins in the aqueous isolation process, which may be otherwise be retained in dry fractionation [48,53]. Both FPR and FPI were relatively rich in lysine. Therefore, both FPR and FPI may be very suitable for blending with certain other plant protein sources such as wheat, which is naturally low in lysine but high in SAAs [66].

#### 3.4.2. In Vitro Protein Digestibility and Trypsin Inhibitor Activity

In vitro protein digestibility (IVPD) and trypsin inhibitor activity (TIA) of faba bean protein ingredients is presented in Table 5. The pepsin digestibility values ranged from 5.4–6.4%, whereas the overall protein digestibility values ranged from approximately 22.2–26.2% (short-term), 25.1–29.9% (medium-term), and 32.9–39.2% (long-term). Pepsin digestibility was significantly higher for FPI compared to both FDH and FPR (*p* < 0.05). FPI generally showed a significantly higher overall protein digestibility at all stages of digestion compared to both FDH and FPR (*p* < 0.05). At medium-term digestion, the FPR exhibited a significantly higher digestibility relative to FDH (*p* < 0.05), but this difference was no longer evident after long-term digestion. The average peptide chain lengths (APCL = 100%/IVPD %) of FDH following pepsin, short-term, medium-term, and long-term digestion were approximately 18.4, 4.5, 4.0, and 3.0 AAs, respectively. The corresponding APCL values of digested FPR were around 17.7, 4.3, 3.7, and 3.0 AAs, whilst for FPI these were about 15.6, 3.8, 3.3, and 2.6 AAs, respectively. Based on both sample and protein mass, the TIA levels were significantly lower in FPI compared to FDH and FPR (*p* < 0.05). These results indicate a positive effect of the aqueous isolation process, with concurrent removal of antinutritional compounds (e.g., trypsin inhibitors) present in faba beans that may otherwise lower the protein digestibility. It is also possible that some remaining intact cell wall in FPR and FDH could have contributed to the lower IVPD.

#### 3.4.3. Vicine and Convicine Analysis

The faba bean ingredients were analyzed for the content of the main antinutritional compounds in faba beans, vicine and convicine, and the results are presented in Table 6. No vicine and convicine was detected in FPI, whereas FDH and FPR had similar total contents of vicine and convicine of 13.9 and 12.5 mg/g sample DM, respectively. The results showed no significant difference in either the individual or total content of vicine and convicine (mg/g sample DM) in FDH and FPR, indicating that vicine and convicine are evenly distributed in the bean cotyledon, and are not concentrated along with the protein when using dry milling (i.e. in FPR). However, when assessing the total contents of vicine and convicine relative to the protein content, a significant decrease was observed from the FDH to the FPR. Importantly, in contrast to dry fractionation, the results demonstrated that it is possible to remove vicine and convicine when using aqueous extraction followed by IEP (i.e., in FPI), as is also supported by previous findings [6,7], where aqueous processing resulted in lower vicine/convicine levels compared to dry processing. 

#### 3.4.4. FODMAP Analysis

Faba beans, and other pulses, are known to be high in FODMAPs, particularly GOS, for example Njoumi et al. [67] reported 3.5% (DM) GOS in raw faba beans. As the cutoff level of GOS that can trigger gastrointestinal symptoms in IBS patients is estimated to be 0.3 g per serving of food [68], consumption of pulses such as faba beans may cause gastrointestinal discomfort. FODMAP levels in FPR, FPI, and FDH for comparison are shown in Table 7, where it can be seen that total GOS was slightly increased by dry fractionation, whereas the isolation process of FPI eliminated most of the GOS present in the starting material. FPR may not be suitable for low FODMAP food formulations except in small amounts, due to its high GOS content. For example, a 200 g serving of food containing only 2.75% protein from FPR would already reach the aforementioned symptom-triggering threshold. FPI, on the other hand, would have to be consumed in extremely large amounts to trigger symptoms and should therefore be suitable for low FODMAP formulations. 

In this case, IEP was an effective method to eliminate FODMAPs from pulses while concentrating the protein. However, when Joehnke et al. [69] compared the FODMAP contents of lentil protein isolates produced using UF or IEP, the former process was found to effectively eliminate FODMAPs, whereas the IEP protein isolate still contained ~50% of the GOS level in the starting material. Perhaps the effectiveness of IEP for GOS removal depends on the input material as well as the process used. Presumably, effective removal of the supernatant (containing dissolved oligosaccharides) after IEP, is the critical step in ensuring low FODMAP content. 

### 3.5. Life Cycle Assessment 

**Figure 11:**
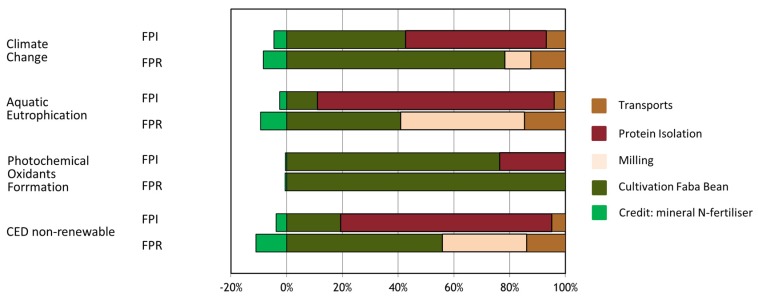
Contributions of main life cycle steps to environmental impact profiles of FPR and FPI; CED = Cumulative primary energy demand.

The environmental performance of FPR and FPI was examined by means of LCA (Table 8). Both climate change results and further indicator results per kg protein are obviously lower for FPR compared to FPI, to a large extent related to the less process energy-demanding milling/air classification process versus the IEP process. The most relevant life cycle steps within the environmental profile of FPR and FPI are the cultivation phase (agriculture) as well as the processing from seeds up to protein isolate powder (in case of FPI) and milling and air classification (in case of FPR), depending on the indicator. The corresponding range in contributions of those life cycle steps to the overall indicator result is illustrated in Figure 11. 

**Figure 12:**
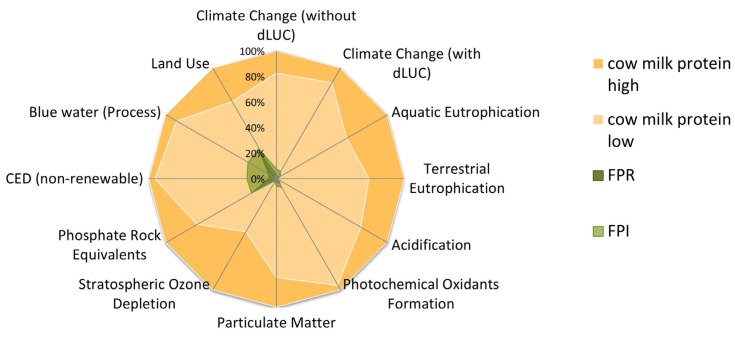
Comparison of environmental impact profiles of FPR and FPI, versus cow’s whole milk powder ranges per kg protein. The highest result is set to 100%; dLUC: direct land use change.

The environmental impact profiles of FPR and FPI were also compared with traditional cow’s milk protein (whole milk powder) ranges on a per kg protein basis (the amount of cow feed per kg milk and share of concentrate versus silage feed are the basis for those examined ranges). FPR and FPI are associated with lower potential environmental impacts than their cow’s milk-based counterpart for all indicators. A comparative illustration of environmental performance (based on all examined indicators) of FPR and FPI versus cow’s milk protein is found in Figure 12.

## 4. Conclusions

Overall, FPR was found to have better techno-functional properties than FPI, particularly with regard to solubility and foaming. FPR was also capable of gelling at lower protein concentrations, and gave stronger gels at the concentrations assessed. However, it is worth noting that functionality may vary considerably for protein isolates depending on the specific process used. SAA content was low in both ingredients, though adequate amounts of lysine were present in both ingredients, which demonstrated good potential as complimentary protein sources for cereals. However, a clear advantage was observed for FPI in terms of eliminating antinutrients and FODMAPs, as well as improving the digestibility of faba bean protein. TIA was significantly lower in FPI, and IVPD was significantly higher when compared to FPR. Vicine/convicine were not detected in FPI. Additionally, in contrast to FPR, FPI is suitable for low FODMAP food formulations. On the other hand, FPR was shown to have a lower environmental impact than FPI per kg protein; however, both ingredients performed considerably better than milk protein in the LCA. Overall, the suitability of IEP and air classified faba bean protein ingredients may depend on the target food application, as well as the intended consumer. In terms of functionality and sustainability, dry fractionation shows promise as a method of protein enrichment. However, if a high protein content and low content of nutritional compounds is desired, IEP or other aqueous processing may be required. Additionally, in the case of patients suffering from IBS or favism, aqueous processing may be necessary to produce suitable ingredients. 

## Figures and Tables

**Table 1:** Nutritional composition of faba bean protein-rich flour (FPR) and faba bean protein isolate (FPI).

(g/100 g) ^1^	FPR	FPI
Moisture	12.2	6.11
Protein	64.1	90.1
Fat	2.43	4.36
SFA	0.40	0.77
MUFA	0.59	1.00
PUFA	1.33	2.38
Ash	4.8	5.2
Total carbohydrate	28.7	0.34
Starch	7.55 ± 0.235	2.48 ± 0.048
Sodium	0.072	0.465

^1^ Solid components are presented as g/100g dry matter. SFA: saturated fatty acids; MUFA: mono-unsaturated fatty acids; PUFA: poly-unsaturated fatty acids; TFA: trans fatty acids; FPI: faba bean protein isolate.

**Table 2:** Particle size parameters and surface hydrophobicity for FPR and FPI.

Particle Size Distribution (μm)	FPR	FPI
D[4,3]	25.4 ± 3.11 ^a^	22.9 ± 2.63 ^a^
D[3,2]	16.1 ± 0.24 ^a^	13.9 ± 0.96 ^b^
Dv(10)	8.65 ± 0.12 ^a^	7.27 ± 0.53 ^b^
Dv(50)	19.3 ± 0.62 ^a^	17.8 ± 1.22 ^a^
Dv(90)	49.1 ± 7.80 ^a^	44.5 ± 4.80 ^a^
Surface hydrophobicity (-)	1208 ± 197 ^a^	2183 ± 370 ^b^

D[4,3]: volume-weighted mean particle diameter; D[3,2]: surface-area weighted mean particle diameter; Dv(10): 10th volume percentile; Dv(50): 50th volume percentile Dv(90): 90th volume percentile. Results are presented as mean ± standard deviation. Values within a row which share a superscript are not significantly different (*p* < 0.05).

**Table 3:** Gelation onset temperature (T_g_), including G’>G” crossover temperature and temperature, where rate of G’ increase reaches 0.5 Pa/°C.

Protein conc.		FPR	FPI
10%	T_g_ (G’>G") (°C)	69.5	63.5
	T_g_ (0.5 Pa/°C) (°C)	84.5	Not applicable (N/A) ^1^
15%	T_g_ (G’>G") (°C)	52.5	N/A ^2^
	T_g_ (0.5 Pa/°C) (°C)	59.5	47.5

^1^ Value not shown for T_g_ (0.5 Pa/°C) FPI 10%, as this rate was not reached until after the initial temperature ramp. ^2^ Value not shown for T_g_ (G’>G") FPI 15% as G’>G” from the beginning.

**Table 4:** Amino acid profiles for FPR and FPI. Results are expressed as g/100 g protein (N×6.25).

Amino Acid	FPR	FPI
Indispensable and conditionally indispensable amino Acids		
Histidine	2.39 ± 0.29	2.49 ± 0.3
Isoleucine	3.73 ± 0.45	4.25 ± 0.52
Leucine	7.10 ± 0.86	8.09 ± 0.98
Lysine	6.34 ± 0.77	6.51 ± 0.79
Methionine	0.60 ± 0.06	0.54 ± 0.04
Cysteine	0.77 ± 0.06	0.62 ± 0.05
Methionine + cysteine	1.37 ± 0.12	1.16 ± 0.09
Phenylalanine	4.13 ± 0.5	4.68 ± 0.57
Tyrosine	3.05 ± 0.37	3.74 ± 0.45
Phenylalanine + tyrosine	7.18 ± 0.87	8.42 ± 1.02
Threonine	3.54 ± 0.43	3.30 ± 0.4
Tryptophan	0.69 ± 0.14	0.74 ± 0.12
Valine	4.14 ± 0.5	4.59 ± 0.56
Dispensable amino acids		
Aspartic acid	10.30 ± 1.25	11.18 ± 1.36
Glutamic acid	16.25 ± 1.97	17.96 ± 2.18
Alanine	3.85 ± 0.47	3.94 ± 0.48
Arginine	10.48 ± 1.27	10.09 ± 1.22
Glycine	3.81 ± 0.46	4.02 ± 0.49
Proline	4.24 ± 0.52	4.45 ± 0.54
Serine	4.87 ± 0.59	5.36 ± 0.65

**Table 5:** In vitro protein digestibility (IVPD) ^1^ and trypsin inhibitor activity (TIA) ^2^ of faba bean ingredients.

	IVPD (%)				TIA	
	Pepsin	Pepsin + Pancreatin				
	1 h	Short-term 1 + 1 h	Medium-term 1 + 3 h	Long-term 1 + 24 h	TIU/mg sample DM	TIU/mg protein
FDH	5.4 ± 0.2 ^b^	22.2 ± 1.0 ^b^	25.1 ± 0.5 ^c^	32.9 ± 0.5 ^b^	1.42 ± 0.20 ^a^	4.33 ± 0.63 ^a^
FPR	5.6 ± 0.3 ^b^	23.1 ± 0.9 ^b^	26.8 ± 0.2 ^b^	33.9 ± 2.1 ^b^	2.34 ± 0.61 ^a^	3.77 ± 0.98 ^a^
FPI	6.4 ± 0.1 ^a^	26.2 ± 0.9 ^a^	29.9 ± 0.7 ^a^	39.2 ± 0.6 ^a^	0.29 ± 0.05 ^b^	0.33 ± 0.05 ^b^

^1^ IVPD (%) according to stage of digestion: pepsin digestibility (1 h) or pepsin + pancreatin overall protein digestibility in the short-term (1 + 1 h), medium-term (1 + 3 h), and long-term (1 + 24 h). IVPD was calculated as the ratio between the concentration of free α-amino groups in the samples and an alanine standard solution, and the results are represented as mean ± SD (*n* = 3). One-way ANOVA followed by Tukey’s post hoc test was conducted within each column and values followed by different letters are significantly different (*p* < 0.05). ^2^ TIA levels (TIU/mg DM) are based on sample mass or protein mass on dry weight basis, and expressed as TIU/mg sample DM or TIU/mg protein DM, respectively. TIA results are presented as mean ± SD (*n* = 3). One-way ANOVA followed by Tukey’s post hoc test was conducted within each column and values followed by different letters are significantly different (*p* < 0.05).

**Table 6:** Content of the antinutritional compounds vicine and convicine in faba bean ingredients ^1^.

Ingredient	Vicine (mg/g DM)	Convicine (mg/g DM)	Total (mg/g sample DM)	Total (mg/g protein)
FDH	8.71 ± 0.65 ^a^	5.20 ± 0.18 ^a^	13.91 ± 0.67 ^a^	37.89 ± 1.83 ^b^
FPR	7.97 ± 1.93 ^a^	4.58 ± 1.23 ^a^	12.54 ± 2.29 ^a^	18.64 ± 3.40 ^a^
FPI	n.d.	n.d.	n.d.	n.d.

^1^ Results are presented as mean ± SD (*n* = 3) on a dry weight basis. Values followed by different letters are significantly different (*p* < 0.05). n.d.= not detected.

**Table 7:** FODMAP content of faba bean ingredients.

FODMAP Category	Carbohydrate	FDH	FPR *	FPI *
Mono-/Disaccharides ^b,c^	Glucose	0.06 ± 0	0.13 ± 0	0.01 ± 0
	Fructose	n.d.	0.09 ± 0	0.02 ± 0
	Excess Fructose ^d^	-	-	-
Polyols ^b^	Xylitol	n.d.	n.d.	n.d.
	Sorbitol	n.d.	n.d.	n.d.
	Mannitol	n.d.	n.d.	n.d.
	∑Polyols	n.d.	n.d.	n.d.
Oligosaccharides	Raffinose/Stachyose ^b^	1.37 ± 0.01	1.42 ± 0.01	0.03 ± 0
	Verbascose ^b^	2.56 ± 0.11	3.45 ± 0.01	0.05 ± 0
	∑GOS	3.93	4.87	0.08
	Total fructan ^e^	n.d.	n.d.	n.d.

^a^ extractions carried out in duplicates and measured via HPAEC-PAD, results referred to dry matter (DM). ^b^ n.d., not detected or levels below 0.005 g/100 g DM. ^c^ no lactose detected in the ingredients. ^d^ EF, excess fructose = glucose–fructose. ^e^ n.d., not detected in means of no significant difference in sucrose values and fructose values, determined from difference of assay A and B in fructan determination, or levels below 0.1 g/100 g DM. * data as reported by Ispiryan et al. [29]

**Table 8:** Environmental impact profile of FPR and FPI, with environment impact potentials expressed per kg protein.

Environmental Indicator	FPR	FPI
Environmental impact potentials (life cycle assessment)		
Climate change (kg CO_2_-e/kg protein)	1.07	3.35
Aquatic eutrophication (g PO_4_-e/kg protein)	4.98	6.53
Terrestrial eutrophication (g PO_4_-e/kg protein)	0.52	1.16
Acidification (g SO_2_-e/kg protein)	6.95	17.3
Photochemical oxidant formation (g O_3_-e/kg protein)	1.26	2.41
Fine particulate matter (g PM2.5-e/kg protein)	5.56	13.9
Stratospheric ozone depletion (mg CFC11-e/protein)	12.3	13.8
Additional indicators at the inventory level (LCI):		
Phosphorus use (g/kg protein)	215	215
Cumulative energy demand, non-renewable (MJ/kg protein)	13.7	54.9
Blue water (process) (kg/kg protein)	13.4	47.7
Land use (m^2^/kg protein)	9.22	9.28
